# Samarium-153-EDTMP (Quadramet^®^) with or without vaccine in metastatic castration-resistant prostate cancer: A randomized Phase 2 trial

**DOI:** 10.18632/oncotarget.10883

**Published:** 2016-07-28

**Authors:** Christopher R. Heery, Ravi A. Madan, Mark N. Stein, Walter M. Stadler, Robert S. Di Paola, Myrna Rauckhorst, Seth M. Steinberg, Jennifer L. Marté, Clara C. Chen, Italia Grenga, Renee N. Donahue, Caroline Jochems, William L. Dahut, Jeffrey Schlom, James L. Gulley

**Affiliations:** ^1^ Laboratory of Tumor Immunology and Biology, Center for Cancer Research, National Cancer Institute, National Institutes of Health, Bethesda, MD, USA; ^2^ Genitourinary Malignancies Branch, Center for Cancer Research, National Cancer Institute, National Institutes of Health, Bethesda, MD, USA; ^3^ Rutgers Cancer Institute of New Jersey, New Brunswick, NJ, USA; ^4^ University of Chicago Medicine, Chicago, IL, USA; ^5^ Biostatistics and Data Management Section, National Cancer Institute, National Institutes of Health, Bethesda, MD, USA; ^6^ National Institute of Biomedical Imaging and Bioengineering, National Institutes of Health, Bethesda, MD, USA; ^7^ Current affiliation: University of Kentucky College of Medicine Lexington, KY, USA

**Keywords:** therapeutic vaccine, radionuclide, prostate cancer, Quadramet^®^, cancer immunotherapy

## Abstract

PSA-TRICOM is a therapeutic vaccine in late stage clinical testing in metastatic castration-resistant prostate cancer (mCRPC). Samarium-153-ethylene diamine tetramethylene phosphonate (Sm-153-EDTMP; Quadramet^®^), a radiopharmaceutical, binds osteoblastic bone lesions and emits beta particles causing local tumor cell destruction. Preclinically, Sm-153-EDTMP alters tumor cell phenotype facilitating immune-mediated killing. This phase 2 multi-center trial randomized patients to Sm-153-EDTMP alone or with PSA-TRICOM vaccine. Eligibility required mCRPC, bone metastases, prior docetaxel and no visceral disease. The primary endpoint was the proportion of patients without radiographic disease progression at 4 months. Secondary endpoints included progression-free survival (PFS), overall survival (OS), and immune responses. Forty-four patients enrolled. Eighteen and 21 patients were evaluable for the primary endpoint in Sm-153-EDTMP alone and combination arms, respectively. There was no statistical difference in the primary endpoint, with two of 18 (11.1%) and five of 21 (23.8%) in Sm-153-EDTMP alone and combination arms, respectively, having stable disease at approximately the 4-month evaluation time point (*P* = 0.27). Median PFS was 1.7 vs. 3.7 months in the Sm-153-EDTMP alone and combination arms (*P* = 0.041, HR = 0.51, *P* = 0.046). No patient in the Sm-153-EDTMP alone arm achieved prostate-specific antigen (PSA) decline > 30% compared with four patients (of 21) in the combination arm, including three with PSA decline > 50%. Toxicities were similar between arms and related to number of Sm-153-EDTMP doses administered. These results provide the rationale for clinical evaluation of new radiopharmaceuticals, such as Ra-223, in combination with PSA-TRICOM.

## INTRODUCTION

Despite recent advances in the treatment of prostate cancer, an estimated 27,540 men died of metastatic disease in the U.S. in 2015 [1]. Approximately 90% of patients with advanced metastatic castration-resistant prostate cancer (mCRPC) have bone lesions, constituting a significant disease burden responsible for morbidity and mortality. Targeting of bone lesions through radionuclides is a reasonable approach in widespread disease. Samarium-153-ethylene diamine tetramethylene phosphonate (Sm-153-EDTMP, Quadramet^®^) is a radiopharmaceutical, which is preferentially absorbed by osteoblastic bone lesions and emits beta particles that cause local tumor cell destruction. It was approved by the United States Food and Drug Administration based on improvement of pain symptoms related to bone lesions [2, 3].

PSA-TRICOM (PROSTVAC, rilimogene galvacirepvec/rilimogene glafolivec) is an active immunotherapeutic cancer vaccine designed to induce activation of T cells specific against prostate-specific antigen (PSA) [4] and has an excellent safety profile [5–8] alone or with external beam radiotherapy [9, 10]. In a randomized placebo-controlled phase 2 study in patients (*n* = 125) with minimally symptomatic or asymptomatic mCRPC, subjects randomized to PSA and a triad of costimulatory molecules (PSA-TRICOM) vaccine had a significantly prolonged median overall survival (OS) compared with those who received empty vector placebo (25.1 vs 16.6 months, hazard ratio (HR) 0.56, stratified log-rank *P* = 0.0061) [8]. Based on those findings, a multi-national phase 3 clinical trial (NCT 01322490) was initiated and has now completed enrollment.

Despite the improvement in OS observed in the randomized phase 2 trial of PSA-TRICOM, there was no difference in progression-free survival (PFS) [8]. This has been observed in other clinical trials involving immunotherapeutics [11, 12], and we have previously hypothesized that a growth rate kinetics model may explain this confounding finding [13–15]. Indeed, the mechanism of action with therapeutic cancer vaccines suggests that a delayed effect should be expected as repeat vaccinations over months are likely to be required to expand antigen-specific T cells, strengthen antigen spreading, and effect an anti-tumor response. This hypothesis has driven the design of phase 2 studies in which we combine therapeutic cancer vaccines with standard agents capable of controlling disease, allowing more time for the generation of a broader, perhaps more clinically relevant, immune response [16, 17].

Radiation has been implicated in immunogenic cell death [18, 19] and immunogenic modulation [20, 21], making it an excellent candidate for combination with immunotherapy, including a randomized phase 3 trial combining radiation therapy with ipilimumab [22]. Our group demonstrated the capability of Sm-153-EDTMP to induce immunogenic modulation, i.e., altering the phenotype of tumor cells to render them more susceptible to T-cell–mediated killing [23]. We thus sought to determine if the use of the palliative Sm-153 EDTMP radionuclide would enhance the therapeutic efficacy of the PSA-TRICOM vaccine. The results reported here support the hypothesis of vaccine radionuclide combination therapy and provide the rationale for the clinical evaluation of a therapeutic radionuclide conjugate such as Ra-223 in combination with PSA-TRICOM vaccination.

## RESULTS

### Patient baseline characteristics

**Figure 1:**
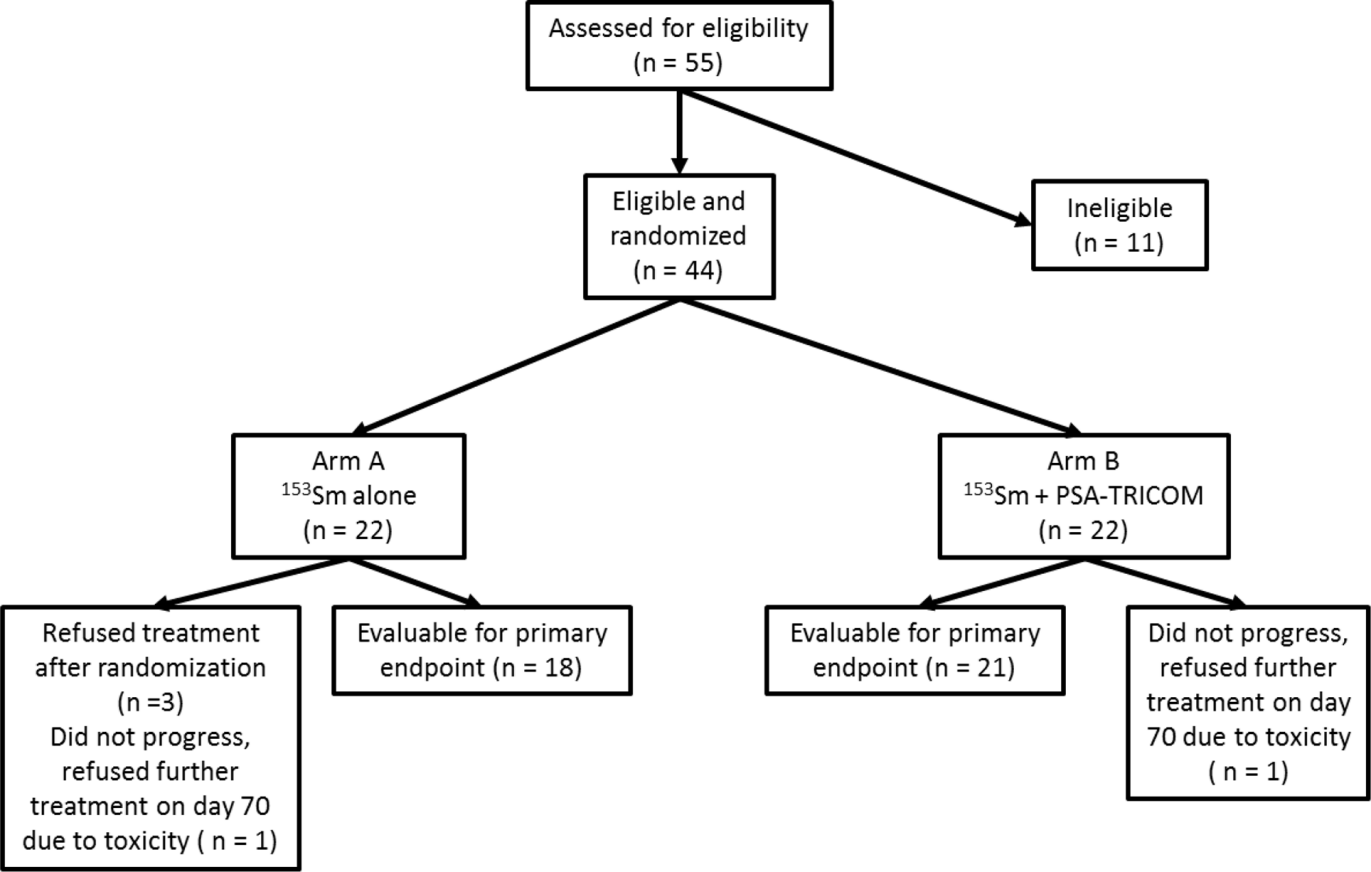
CONSORT diagram The CONSORT diagram illustrates the patients enrolled, randomized, and evaluable for study endpoints.

Forty-four patients were enrolled between February 2007 and May 2012 at National Cancer Institute (*n* = 27), University of Chicago (*n* = 9), and Rutgers Cancer Institute of New Jersey (*n* = 8). Although the trial was designed to enroll 68 patients, the study was ended early due to poor accrual. Twenty-two patients were randomly assigned to each arm. Four patients in arm A and one patient in arm B were not evaluable (Figure 1). Baseline demographics and known prognostic variables were similar between the two groups (Table 1).

**Table 1:** Baseline characteristics

Characteristic	Sm-153 (A) *n* = 22	Sm-153 + PROSTVAC (B) *n* = 22	*p*-values*
Subjects requiring narcotic pain control	11/19 (58%)	8/22 (36%)	*P* = 0.22
Median (range)			
Gleason score	8 (6–9)	7.5 (5–10)	*P* = 0.81
Age	64.5 (50–80)	69.2 (52–86)	*P* = 0.058
PSA on study	259.1 (22.2–1856)	313.5 (4.9–4708)	*P* = 0.78
ECOG performance status	1 (0–1)	1 (0–2)	*P* = 0.52^/*P* = 0.28#
Median days since prior chemotherapy	57.5 (11–301)	86.5 (21–818)	*P* = 0.30
Lactate dehydrogenase (serum)	254.5 (175–353)	200 (115–962)	*P* = 0.31
Hemoglobin	11.2 (8.8–13.2)	11.1 (7.0–14.8)	*P* = 0.80
Alkaline phosphatase	177 (90–725)	121.5 (52–661)	*P* = 0.041

ECOG, Eastern Cooperative Oncology Group, NCI; PSA, prostate-specific antigen.

* *P*-values determined by exact Wilcoxon rank sum test except where noted

^ -Fisher's exact test

# = Cochran-Armitage test for trend).

### Safety

The majority of adverse events were attributable to Sm-153-EDTMP. Grade 3 and 4 events were similar between both arms when controlled for the number of doses of Sm-153-EDTMP given in each arm (Supplementary Table S1). Hematologic toxicities were most commonly associated with Sm-153-EDTMP. The most common adverse event attributed to vaccine was injection site reaction, which was transient, self-limited, and did not exceed grade 2 in all cases (34 events in 10 distinct patients).

### Clinical outcomes

**Figure 2:**
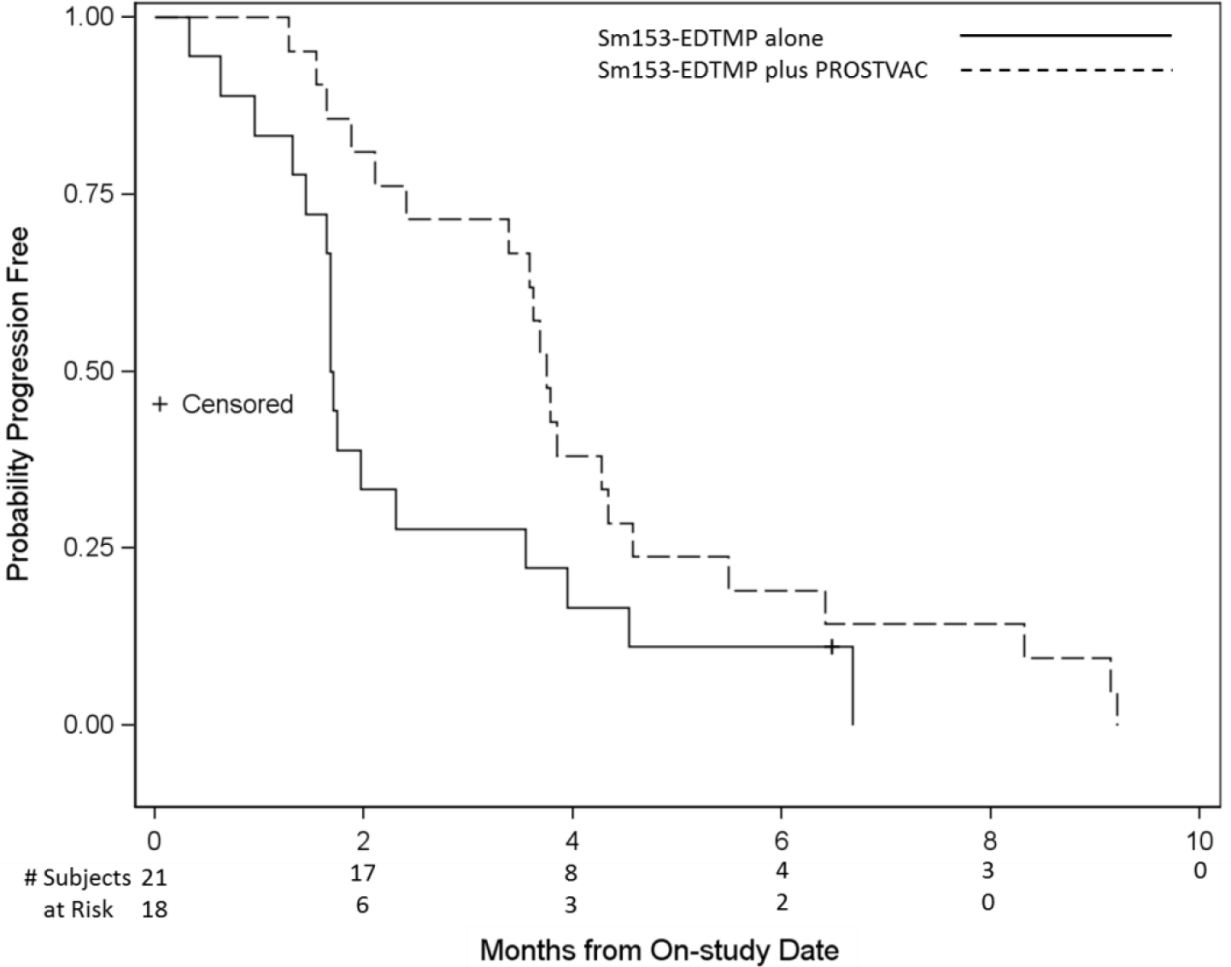
Progression-free survival (PFS) Kaplan-Meier curve of PFS of patients on arm A (Sm-153-EDTMP alone) vs arm B (Sm- 153-EDTMP in combination with PSA-TRICOM vaccine). Median PFS in arm B was 3.7 months compared with 1.7 months in arm A, *P* value 0.021 (one-tailed), 0.041 (two-tailed).

**Figure 3:**
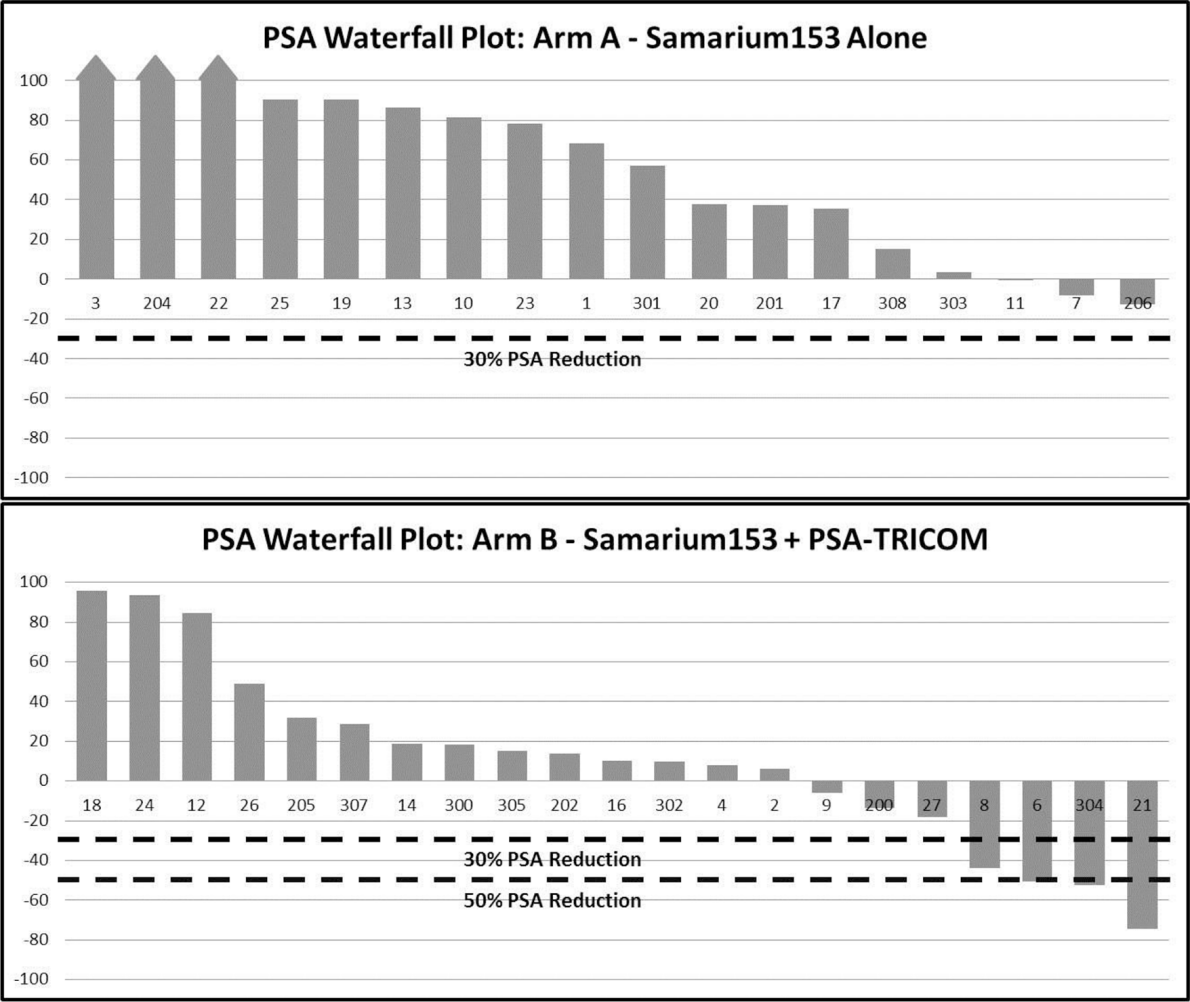
Serum PSA Waterfall plot Greatest percentage change in PSA for each patient while on treatment. (**A**) (Sm-153-EDTMP alone, *n* = 18 evaluable.) No patient achieved a PSA decline of > 30%. (**B**) (Sm-153-EDTMP in combination with PSA-TRICOM vaccine, *n* = 21 evaluable.) 4/21 patients achieved > 30% PSA decline and 3/21 achieved > 50% PSA decline.

Of 44 patients enrolled, 18 and 21 were evaluable for the primary endpoint in arms A and B, respectively (Figure 1). There was no statistical difference in the primary endpoint, with two of 18 (11.1%) in arm A and five of 21 (23.8%) in arm B having stable disease at approximately the 4-month evaluation time point (*P* = 0.27). Based on the same 39 patients evaluated for the 4-month endpoint, however, the median PFS was 1.7 and 3.7 months in arms A and B, respectively (*P* = 0.021, one-tailed, *P* = 0.041, two-tailed, with HR = 0.51, *P* = 0.046, 95% CI on HR: 0.26–0.99; Figure 2). If, in addition, we include the other five patients who were inevaluable for the 4-month PFS evaluation, resulting in 44 total patients, the median PFS remains 1.7 and 3.7 months, but now with inevaluable patients, one-tailed *P* = 0.025 and two-tailed *P* = 0.051, with HR = 0.52, two- tailed *P* = 0.056, 95% CI on HR: 0.27–1.02 (data not shown). No PSA serum response was observed in arm A. In arm B, four of 21 (19%) evaluable patients had PSA decline ≥ 30% and three of 21 (14.3%) had PSA decline ≥ 50% (Table 2, Figure 3). No statistical difference in OS was observed between the two arms, with a median of 8.1 and 9.2 months, respectively, in arms A and B (HR = 0.71, 95% CI on HR: 0.37–1.35 inevaluable 0.30).

**Table 2:** Clinical outcomes

	Sm-153 (A)	Sm-153 + PROSTVAC (B)	Statistics for comparison
Evaluable patients	*n* = 18	*n* = 21	
PFS			
Fraction at 4 months	2/18 (11.1%)	5/21 (23.8%)	*P* = 0.27
Median PFS months	1.7	3.7	*P* = 0.021 (one-tailed), 0.0412 (two-tailed) HR 0.51, *P* = 0.046
Pt # confirmed PSA decline			
≥ 30%	0	4/21 (19.0%)	
≥ 50%	0	3/21 (14.3%)	

PFS, progression-free survival; PSA, prostate-specific antigen.

### Immune assays

Sufficient PBMCs were available pre- and post-therapy (approximately 60 days) from eight patients treated with Sm-153-EDTMP alone and 10 patients administered Sm-153-EDTMP plus vaccine to measure PSA-specific CD4+ and CD8+ T-cell responses. The FACS-based assay for T cells expressing type I cytokines IFN-γ, IL-2, TNF-α, and/or CD107a (a marker for lytic potential) is described in detail in the Methods section. Two of eight (25%) patients in the Sm-153-EDTMP alone arm developed measurable PSA-specific responses following therapy, while six of 10 (60%) in patients treated with Sm-153-EDTMP plus vaccine developed PSA-specific T cells (*P* = 0.19 by Fisher's exact test; Table 3). Four out of 18 patients had some level of PSA-specific T cells prior to therapy; of those, only one out of four went on to develop enhanced PSA-specific T-cell responses post–Sm-153-EDTMP plus vaccine therapy. CD107a positivity is known to be a marker of a T cell with lytic potential. One of eight patients in the Sm-153-EDTMP alone arm developed CD107a+, CD4+ or CD8+ T cells post-therapy, while five of 10 patients in the combination arm developed CD107a+, CD4+ or CD8+ T cells post-therapy (*P* = 0.15; Table 3).

**Table 3:** Sm-153 ± PROSTVAC trial

PSA-specific T-cell responses post- vs. pre-treatment											
	CD4						CD8				# of Responses^a^
	PT	CD107a	IFN-γ	IL2	TNF		CD107a	IFN-γ	IL2	TNF	
Quadramet Alone	11	0	0	0	0		0	0	56	186	2/8 (25%)
	13*	0	0	0	0		0	0	0	3	
	20*	0	0	0	0		0	0	0	0	
	22	154	0	0	0		**1427**	0	0	**274**	
	25	146	0	0	0		0	248	30	**630**	
	3	0	0	0	0		0	0	14	0	
	5	0	0	0	0		0	0	0	25	
	10	0	6	0	0		0	21	0	0	
Quadramet + PROSTVAC	2	0	**786**	**0**	**374**		**5269**	**453**	**0**	323	6/10 (60%)
	8	58	**345**	0	245		**633**	136	0	35	
	12	0	0	0	0		58	0	0	0	
	18	0	0	75	**402**		0	0	0	35	
	21	0	45	0	0		0	214	0	0	
	24*	0	0	149	181		**1242**	0	8	179	
	14	**821**	0	0	0		0	0	0	0	
	16	0	0	0	**0**		0	0	9	0	
	26	**815**	0	0	0		149	0	0	0	
	27*	0	0	0	0		0	0	0	0	

a Cytokine or CD107a in CD4 or CD8.

* Patients displayed pre-existing PSA-specific T-cell responses.

Numbers in bold are those positive post- vs. pre-vaccination.

Absolute # of CD4 or CD8 producing cytokine or CD107a^+^/1 × 10^6^ cells plated at start of *in vitro* stimulation.

Sufficient PBMCs were available from a subset of patients treated at the NCI that received Sm-153-EDTMP alone (*n* = 7) or Sm-153-EDTMP plus vaccine (*n* = 9) for the analysis of 110 different immune cell subsets (Supplementary Table S2) at pre- and post-therapy (∼day 60). There were no significant changes in the nine standard immune cell subsets evaluated, including CD4+ T lymphocytes, CD8+ T lymphocytes, regulatory T cells (Tregs), natural killer (NK) cells, NK T cells, B lymphocytes, conventional dendritic cells (DCs), plasmacytoid DCs, or myeloid derived suppressor cells (MDSCs) pre- vs post-therapy in patients in either arm (Supplementary Table S3). Evaluation of 101 additional subsets relating to the maturation/function of the nine standard immune cells identified some trends (Supplementary Table S4). Notably, patients receiving Sm-153-EDTMP alone, but not Sm-153-EDTMP plus vaccine, displayed an increase in several MDSC subsets, including those expressing PDL1 (PDL1 + MDSC, *P* = 0.016) that are reported to have a suppressive function [24]. In contrast, patients treated with Sm-153-EDTMP plus vaccine displayed trends mainly in T lymphocytes in relation to memory and activation status not seen with Sm-153-EDTMP alone. Both CD4 and CD8 cells with an activated phenotype (ICOS+PDL1+CD4, *P* = 0.020, and PDL1+CD8, *P* = 0.039) were decreased, while central memory CD4 were increased (*P* = 0.020), which would be expected to be immune potentiating.

**Figure 4:**
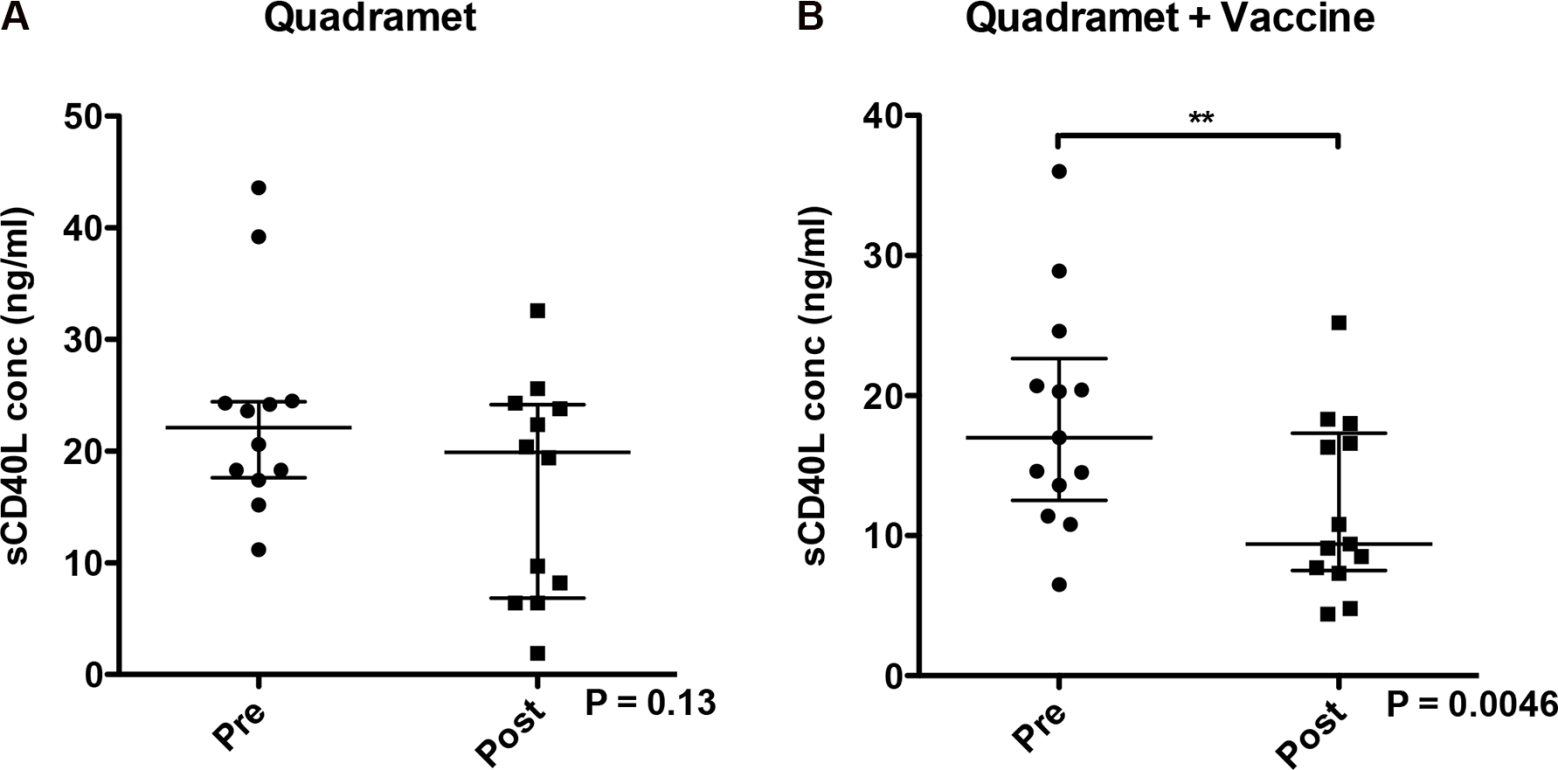
Serum levels of sCD40L Serum levels of sCD40L decreased significantly after treatment with Sm-153-EDTMP in combination with vaccine. Soluble CD40L in serum was measured by ELISA before and during therapy. (**A**) Patients treated with Sm-153-EDTMP alone. (**B**) Patients treated with Sm153-EDTMP and vaccine. Dot plots show results for individual patients, the median and the interquartile range. Wilcoxon signed rank test. **P = 0.0046.

Soluble CD40L in sera has been implicated as a negative prognostic indicator in some cancers, and is associated with decreased immune cell function. The serum levels of sCD40L were measured before and during therapy in both arms. There was no overall change in patients receiving Sm-153-EDTMP alone (Figure 4A), but a trend toward decreased sCD40L (*P* = 0.0046) in patients receiving Sm-153-EDTMP in combination with vaccine (Figure 4B), indicating a potential enhancement in immune cell function by the vaccine.

## DISCUSSION

The trial presented here was designed to evaluate whether the addition of vaccine to Sm-153-EDTMP could improve PFS at 4 months. Despite the poor accrual of this trial due to newly available active oral agents in the post-docetaxel setting which limited the interpretability of the primary endpoint, we found a significant increase in PFS. In addition to the improvement in PFS, we observed evidence of serum PSA declines in the combination arm that were not seen in the Sm-153-EDTMP alone arm.

It is important to put the PFS findings from this study in the context of contemporary studies conducted in mCRPC patients previously treated with docetaxel that accrued prior to the approval of enzalutamide and abiraterone. For example, the phase 3 trial that led to the approval of cabazitaxel demonstrated a median PFS of 1.4 months for the control (mitoxantone and prednisone) compared to 2.8 months in median PFS for patients treated with cabazitaxel. Based on these values, it would seem that the Sm-153-EDTMP control group patients in this study (PFS 1.7 months) are similar to other patients treated with palliative therapies at that time. The resulting improvement in PFS compared to a control group with median PFS similar to that of a historical control is encouraging and requires further exploration.

Despite the inability to complete accrual of this trial, which was likely attributable to the recent approval of a number of new agents for mCRPC, we observed a suggestion of clinical benefit with the combination of Sm- 153-EDTMP and PSA-TRICOM compared with Sm-153-EDTMP alone without the burden of additional toxicity. These results support the hypothesis that therapeutic cancer vaccines may slow tumor growth over time and treatment with vaccines in combination with synergistic cytotoxic agents may provide ample time and relative tumor control to convey clinical benefit [17]. We believe this finding to be consistent with the previous finding that vaccine alone does not improve PFS but does improve OS [12] and supports the growth rate kinetics model previously created based on the clinical trial data using PSA-TRICOM alone [15].

A reasonable concern related to the use of radiotherapeutic agents in combination with immunotherapy is the potential to cause destruction of immune cell subsets and prevent immune-mediated anti-tumor activity. However, previous work from our group demonstrated no increase in apoptosis of immune cells in the tumor microenvironment of mice treated with vaccine and a radiolabeled monoclonal antibody compared with vaccine alone [25], indicating memory T cells are more resistant to radiation induced apoptosis than naïve T cells, similar to the findings of other groups [26]. The immune analyses reported here indicate that Sm-153-EDTMP did not have a deleterious effect on any of the 110 immune cell subsets in PBMCs in either arm, and patients in the vaccine arm were still able to mount CD107a+ PSA-specific T cells, providing evidence of lytic potential. Additionally, there was a trend toward increased number of PSA-specific T cells generated in the combination arm and a trend toward decreased sCD40L, despite the small number of evaluable patients for these analyses. Careful selection of the agent for use in combination is essential. We selected Sm-153-EDTMP based on previous preclinical data, which demonstrated the capacity of Sm-153-EDTMP to induce “immunogenic modulation” in tumor cells, making them more amenable to T-cell–mediated killing. The lack of PSA responses with Sm-153-EDTMP alone compared with PSA responses when used in combination (Table 2, Figure 3) further suggests activity of the combination.

## MATERIALS AND METHODS

### Patient eligibility

Subjects were required to have mCRPC with bone metastases as determined by CT and/or bone scan with pathology diagnosis confirmed by the Laboratory of Pathology, National Institutes of Health. No visceral metastases were allowed, but small, asymptomatic lymph nodes were allowed. Previous treatment with docetaxel was required, and there were no limits on the number of prior chemotherapy or hormonal therapy regimens allowed for enrollment. Prior treatment with Sm-153-EDTMP was not allowed. Subjects were required to remain on testosterone-suppressing therapy unless they were surgically rendered castrate. Subjects were required to be ≥ 18 years of age, have acceptable hematologic parameters and organ function, have an Eastern Cooperative Oncology Group (ECOG) performance status of ≤ 2, have no other malignancies within 12 months, or significant medical illnesses or autoimmune diseases. No systemic steroid use was allowed within 2 weeks of enrollment. Due to vaccinia vector used in priming, subjects with a history of prior allergy or severe reaction to vaccinia-based vaccination or an open skin wound were also excluded. This study is registered with ClinicalTrials.gov, number NCT00450619.

### Trial design and treatment

Subjects were randomized 1:1 to receive Sm- 153-EDTMP alone (arm A) or in combination with PSA-TRICOM (arm B). All subjects were treated with Sm- 153-EDTMP per standard dosing, 1 mCi/Kg IV over 1 minute, which was given on day 8 after randomization (to allow for ordering radionuclide) and then every 12 weeks if adequate hematologic recovery had occurred. In arm B, subjects also received subcutaneous injections of rV-PSA-TRICOM on day 1 (2 × 10^8^ plaque-forming units (PFU)) and then rF-PSA-TRICOM (1 × 10^9^ PFU) on days 15 and 29, and every 28 days thereafter. Baseline imaging, consisting of bone scan and CT chest/abdomen/pelvis, was performed prior to randomization and repeated at 2 months, 4 months, and then every 3 months thereafter. To account for scintigraphic “flare” phenomenon described with effective treatment [28], the repeat bone scan at 2 months was used as the baseline if any changes occurred and for comparison to the 4-month restaging scan. Disease progression was defined using Prostate-Specific Antigen Working Group criteria [29]. Adverse events were monitored using Common Terminology Criteria for Adverse Events (CTCAE) version 3.0. The treatment protocol was approved by the National Cancer Institute, University of Chicago, and Rutgers Cancer Institute of New Jersey, Institutional Review Boards and subjects were enrolled at each institution.

### Statistical considerations

This was conducted as a randomized two-arm trial, with the primary endpoint being a comparison of the proportion of patients on each arm with progression at a 4-month evaluation. The study was designed to enroll 34 evaluable patients per arm (68 total) to provide 80% power to detect a difference between 15% and 40% PFS at 4 months in arms A and B, respectively, using a one-tailed alpha = 0.10 Fisher's exact test, following the principles of a phase 2.5 study design [30]. Secondary endpoints included an overall analysis of PFS, OS, and changes in serum PSA, comparison of toxicity, and evaluation of antigen-specific T-cell activation. Analyses of PFS were performed using the Kaplan-Meier method, beginning at the on-study date through the date of progression or last follow-up without progression. The hazard ratio (HR) comparing the two curves was estimated using Cox regression analysis. Comparisons of pre-treatment and post-treatment immune parameters were performed with a Wilcoxon signed rank test. Except as noted, all *P* values are two-tailed, and all are presented without adjustment for multiple comparisons.

Baseline characteristics using all available data from the 22 patients randomized per arm were compared between the two arms using an exact form of the Wilcoxon rank sum test for continuous parameters. The fraction of patients requiring narcotics, as well as the fraction of patients with ECOG performance status 0 vs. 1–2, were compared between the two arms using Fisher's exact test. The actual distribution of ECOG performance status values was also compared between the arms using a Cochran-Armitage test for trend.

### Randomization and masking

Patients were randomized centrally, without stratification, using a locally-written SAS software program to generate a random 1:1 sequence of assignments to treatment, using variable block sizes (2 or 4), with parameters for assignment determined by the study statistician (SMS). The randomization assignment sheets were maintained confidentially in a central registration office, which enrolls the patients; the treatment assignment for a given patient was only disclosed to the study research team by a member of the central registration staff after confirming full eligibility.

### Immune assays

Peripheral blood mononuclear cells (PBMCs) were isolated, cryopreserved, and assessed for the frequency of immune cell subsets by multi-parametric flow cytometry as previously described [17]. One vial of PBMCs from patients before therapy (baseline) and at approximately day 60 of therapy was defrosted and stained in panels using antibodies (listed in Supplementary Table S5) to identify nine standard immune cell subsets including CD4+ and CD8+ T lymphocytes, Tregs, NK cells, NK-T cells, B lymphocytes, conventional and plasmacytoid DCs, and MDSCs, and 101 additional subsets relating to the maturation/function of the standard subsets (Supplementary Table S4). For all flow cytometry experiments, upto 3 × 10^5^ live events were acquired with a BD LSR-II flow cytometer. Data were analyzed with FlowJo V.9.7 for Macintosh (TreeStar, Ashland, OR) with fluorescence minus one controls used for gating, and non-viable cells excluded.

Antigen-specific responses were assessed by intracellular cytokine staining (ICS) following a period of *in vitro* stimulation (IVS) of PBMCs with overlapping 15-mer peptide pools encoding the tumor-associated antigen (TAA) PSA. The PSA peptide pool contained a previously identified agonist epitope [31]; pools encoding for HLA and CEFT (a mixture of CMV, EBV, Flu, and tetanus toxin) served as negative and positive controls, respectively. PBMCs from patients before and 60 days following therapy were stimulated in an IVS and stained with antibodies (listed in Supplementary Table S6) to identify the absolute number of CD4+ or CD8+ lymphocytes producing cytokine (IFN-γ, TNF, or IL-2) or positive for CD107a as previously described [32]. The background signal (obtained with the HLA peptide pool) and values obtained prior to therapy were subtracted from those obtained post-therapy. Values >250 were scored as positive for TAA-specific immune response following therapy if they were also at least 2-fold greater than that obtained with HLA.

The serum levels of sCD40L were determined by the human sCD40L Platinum ELISA kit (eBioscience, San Diego, CA) according to the manufacturer's instructions and as previously described [33].

## CONCLUSIONS

Sm-153-EDTMP in combination with PSA-TRICOM appears to lead to clinically meaningful improvement in PFS and is associated with trends to improved PSA declines and PSA-specific T-cell responses compared with Sm-153-EDTMP alone. This study has limitations due to the small number of patients enrolled, but it did achieve the goal of providing the rationale for and estimates for statistical assumptions for a larger, randomized study employing a modern radiopharmaceutical in combination with PSA-TRICOM. Ra-223, one such modern radiopharmaceutical that was more recently approved for the treatment of mCRPC, may be a better selection for use in combination with immunotherapy, due to its demonstrated effect on overall survival [27], compared with Sm-153-EDTMP, which has greater hematologic toxicity and only demonstrated a palliative effect in clinical trials. Ongoing preclinical testing will determine if Ra-223 has similar immunomodulatory effects to support its use in combination with immunotherapy in future clinical trials.

## SUPPLEMENTARY MATERIALS TABLES


