# Supplementary material for: Samarium-153-EDTMP (Quadramet®) with or without vaccine in metastatic castration-resistant prostate cancer: A randomized Phase 2 trial
Source: Oncotarget. 2016 Jul 28;7(42):69014–23. doi: 10.18632/oncotarget.10883 (PMC5340090; doi:10.18632/oncotarget.10883)
Supplement: Supplementary file 1 [file oncotarget-07-69014-s001.pdf]

Samarium-153-EDTMP (Quadramet®) with or without vaccine in metastatic castration-resistant prostate cancer: A randomized phase 2 trial

Supplementary Materials

Supplementary Table S1: Adverse events, grade 3 and 4 only, all attributions

	Cohort A		Cohort B	
	Patients (% of total patients)	Events (events per dose Sm = 153)	Patients (% of total patients)	Events (events per dose Sm = 153)
**Grade 3**				
Hematologic	10 (45.5%)	22 (1.0)	13 (59.1%)	43 (1.1)
Anemia	7 (31.8%)	10 (0.4)	4 (18.2%)	6 (0.15)
ALC decreased	2 (9.1%)	2 (0.08)	3 (13.6%)	5 (0.13)
ANC decreased	2 (9.1%)	2 (0.08)	4 (18.2%)	9 (0.23)
Platelets decreased	3 (13.6%)	7 (0.28)	8 (36.4%)	13 (0.33)
WBC decreased	0 (0%)	1 (0.04)	5 (22.7%)	9 (0.23)
INR decreased	0 (0%)	0 (0)	1 (4.5%)	1 (0.03)
Gastrointestinal	3 (13.6%)	6 (0.24)	4 (18.2%)	6 (0.15)
Constitutional	3 (13.6%)	3 (0.12)	4 (18.2%)	5 (0.13)
Musculoskeletal	4 (18.2%)	3 (0.12)	5 (22.7%)	8 (0.21)
Electrolyte/Metabolic	1 (4.5%)	1 (0.04)	3 (13.6%)	3 (0.08)
Genitourinary	1 (4.5%)	1 (0.04)	0 (0%)	0 (0)
Infection	0 (0%)	0 (0)	1 (4.5%)	1 (0.03)
Dental	0 (0%)	0 (0)	1 (4.5%)	1 (0.03)
**Grade 4**				
Hematologic	2 (9.1%)	2 (0.08)	3 (13.6%)	3 (0.08)
**Grade 5**				
Death	1 (4.5%)	1 (0.04)	0 (0%)	0 (0)

Supplementary Table S2: Flow-cytometry analysis of PBMC immune subsets: 25 markers, 110 subsets

1. **CD4**: Helper T lymphocytes (28 subsets)
2. **CD8**: Cytotoxic T lymphocytes (25 subsets)
    - **Maturation status of T lymphocytes (in CD4 and CD8)**:
        - **Naïve**: CD45RA$^+$ CCR7$^+$
        - **Central Memory**: CD45RA$^-$ CCR7$^+$
        - **Effector Memory**: CD45RA$^-$ CCR7$^-$
        - **Terminal (EMRA)**: CD45RA$^+$ CCR7$^-$
    - **T lymphocyte markers (in CD4 and CD8)**:
        - **CTLA-4**: inhibition
        - **PD-1**: activation/inhibition
        - **PD-L1**: activation/cross-inhibition
        - **TIM-3**: inhibition
        - **ICOS**: activation (only on CD4)
3. **Tregs**: Regulatory T lymphocytes (CD4$^+$ CD25$^+$ FoxP3$^+$ CD127$^-$) (6 subsets)
    - **CD45RA**: Tregs highly expandable *in vitro*
    - **CTLA-4**: Treg suppression
    - **ICOS**: Treg suppression
    - **PD-1**: activation/inhibition
    - **PD-L1**: cross-inhibition
4. **B lymphocytes**: CD19$^+$ (3 subsets)
    - **PD-1**: activation/inhibition
    - **PD-L1**: cross-inhibition
5. **NK**: Natural killer cells (CD56$^+$ CD3$^-$) (20 subsets)
    - **CD16$^+$ CD56^{br}**: Functional intermediate, lytic and cytokine production
    - **CD16$^+$ CD56^{dim}**: Mature NK, cytokine production
    - **CD16$^-$ CD56^{br}**: Immature, abundant in human placenta
    - **CD16$^-$ CD56^{dim}**: non-lytic, non-cytokine production
    - **TIM-3**: activation
    - **PD-1**: activation/inhibition
    - **PD-L1**: cross-inhibition
6. **NK-T**: CD56$^+$ CD3$^+$ (4 subsets)
    - **TIM-3**: activation
    - **PD-1**: activation/inhibition
    - **PD-L1**: cross-inhibition
7. **cDCs (conventional DCs)**: CD3$^-$CD56$^-$CD11c^{br}CD303$^-$CD141$^-$ (4 subsets)
8. **pDCs (plasmacytoid DCs)**: CD3$^-$CD56$^-$CD1c$^-$CD303$^+$ (4 subsets)
    - **Markers of DC activation**
        - **TIM-3**: inhibition
        - **PD-1**: activation/inhibition
        - **PD-L1**: cross-inhibition
9. **MDSCs**: Myeloid-derived suppressor cells (CD11b$^+$ HLA-DR$^{low/-}$ CD33$^+$) (16 subsets)
    - **CD14**: Common Myeloid Marker (high in monocytes, dim in granulocytes)
    - **CD15**: Granulocyte marker
    - **CD68**: Monocyte/macrophagemarker
    - **PD-1**: activation/inhibition
    - **PD-L1**: cross-inhibition

Subsets analyzed: 9 standard immune cell subsets, PD-L1 and PD-1 in standard subsets, and 83 additional subsets relating to maturation/function.

Supplementary Table S3: Standard immune cell subsets

Subset (% of PBMC)	Interquartile range of pre	Sm-153 Alone (*n* = 7)				Sm-153 + PROSTVAC (*n* = 9)			
		Median		*P* value (unadjusted)	*P* value (adjusted)	Median		*P* value (unadjusted)	*P* value (adjusted)
		Pre	Post			Pre	Post		
CD4	24.2–31.2	27.5	29.1	> 0.999	> 0.999	27.6	33.8	0.2031	> 0.999
CD8	15.2–25.1	23.2	29.0	0.0781	0.702	20.4	18.1	0.6523	> 0.999
Treg	2.02–3.11	2.53	2.73	0.3750	> 0.999	2.41	2.54	0.5703	> 0.999
NK	7.6–12.4	8.5	8.6	0.1563	> 0.999	11.1	8.8	0.8203	> 0.999
NK=T	0.61–1.58	1.141	1.178	0.5781	> 0.999	0.779	1.039	0.6523	> 0.999
B Lymphocyte	2.54–6.18	4.00	3.74	0.8125	> 0.999	5.04	4.96	0.6523	> 0.999
cDC	0.047–0.342	0.124	0.154	0.2969	> 0.999	0.128	0.096	0.8203	> 0.999
pDC	0.174–0.407	0.270	0.201	0.2188	> 0.999	0.198	0.259	> 0.999	> 0.999
MDSC	3.26–9.94	5.26	7.44	0.2969	> 0.999	4.60	8.70	0.2031	> 0.999

Abbrevations: Tregs, regulatory T cells; NK, natural killer cells; NK-T, natural killer T cells; cDC, conventional dendritic cells; pDC, plasmacytoid dendritic cells; MDSCs, myeloid-derived suppressor cells.

Supplementary Table S4: Immune cell subsets relating to maturation/function

		Sm-153 Alone ($n = 7$)				Sm-153 + PROSTVAC ($n = 9$)			
Subset (% of PBMC)	Interquartile range of pre	Median		P value (unadjusted)	P value (adjusted)	Median		P value (unadjusted)	P value (adjusted)
		Pre	Post			Pre	Post		
CD4 ICOS$^+$PDL1$^+$	0.129–0.246	0.166	0.155	0.0469$^+$	> 0.999	0.204	0.124	**0.0195**	0.468
CD4 CM	0.395–1.1202	0.440	0.636	0.0469$^+$	> 0.999	0.769	1.343	**0.0195**	0.468
CD8 PDL1$^+$	0.176–0.305	0.207	0.266	0.5781	> 0.999	0.220	0.170	**0.0391**	0.897
CD8 PDL1$^+$ EM	0.083–0.186	0.097	0.117	0.8125	> 0.999	0.129	0.072	**0.0391**	0.897
NK PD1$^+$ CD16$^-$CD56^{dim}	0.040–0.090	0.052	0.121	**0.0156**	0.288	0.067	0.070	0.4258	> 0.999
MDSC PDL1$^+$	0.503–2.021	1.469	2.665	**0.0156**	0.224	0.534	0.891	0.0977	0.999
mMDSC PDL1$^+$	0.266–1.107	0.421	2.063	**0.0156**	0.224	0.342	0.592	0.0195$^+$	0.273
mMDSC PD1$^+$	0.029–0.151	0.077	0.173	**0.0156**	0.224	0.054	0.136	0.0742	> 0.999

$^+$Not considered a potentially biologically relevant change, because median post value falls within the interquartile range of pre.
Bold indicates significant *P* values.

Supplementary Table S5: Antibodies for assessing frequency of peripheral immune cell subsets

Marker	Conjugate	Clone	Company
CTLA4	FITC	L3D10	BioLegend
PD1	PE	MIH4	BD Bioscience
CCR7	PerCp-Cy5.5	150503	BD Bioscience
PDL1	PE-Cy7	MIH1	BD Bioscience
Tim3	BV421	F38-2E2	BioLegend
CD4	BV605	RPA-T4	BD Bioscience
CD8	APC	RPA-T8	BioLegend
CD45RA	AF700	HI100	BD Bioscience
CD19	APC-H7	SJ25C1	BD Bioscience
ICOS	PerCP-Cy5.5	C398.4A	BioLegend
FoxP3	Pac. Blue	206D	BioLegend
CD25	APC	M-A251	BioLegend
CD127	APC-eF780	eBioRDR5	eBioscience
CD15	FITC	HI98	eBioscience
CD68	PerCp-Cy5.5	Y1/82A	BioLegend
CD14	V450	M0Pg	BD Bioscience
HLA-DR	BV605	L243	BioLegend
CD33	APC	WM53	BioLegend
CD11b	APC-Cy7	ICRF44	BD Bioscience
CD16	FITC	3G8	BD Bioscience
CD303	PerCp-Cy5.5	201A	BioLegend
CD11c	BV605	3.9	BioLegend
CD56	APC	MEM-188	BioLegend
CD141	BV711 (AF700)	1A4	BD Bioscience
CD3	APC Cy7	UCHT1	BioLegend

Supplementary Table S6: Antibodies for assessing antigen-specific responses

Marker	Conjugate	Clone	Company
TNF	PE	MAb11	BD Bioscience
CD4	PerCp-Cy5.5	OKT4	BioLegend
IFNγ	PE-Cy7	4S.B3	BD Bioscience
IL-2	BV521	MQ1-17H12	BioLegend
CD107a	APC	H4A3	BD Bioscience
CD8	AF700	RPA-T8	BD Bioscience
